# YTHDF1 Regulates Tumorigenicity and Cancer Stem Cell-Like Activity in Human Colorectal Carcinoma

**DOI:** 10.3389/fonc.2019.00332

**Published:** 2019-05-03

**Authors:** Yang Bai, Chunxing Yang, Runliu Wu, Lihua Huang, Shenlei Song, Wanwan Li, Peichen Yan, Changwei Lin, Daojiang Li, Yi Zhang

**Affiliations:** ^1^Department of Gastrointestinal Surgery, The Third Xiangya Hospital of Central South University, Changsha, China; ^2^Center for Medical Experiments, The Third Xiangya Hospital of Central South University, Changsha, China

**Keywords:** CRC, YTHDF1, CNV, tumorigenicity, CSC, Wnt/β-catenin pathway

## Abstract

YTH N6-methyladenosine (m6A) RNA binding protein 1 (YTHDF1) is a core factor in RNA methylation modification. Recent studies have shown that m6A is closely related to multiple tumors, thus YTHDF1 may also play a role in tumorigenesis. This study, aimed to explore the role of YTHDF1 in the colorectal cancer (CRC). In this study, we identified YTHDF1 as being highly expressed at the mRNA and protein levels in TCGA, GEO CRC and primary CRC. Furthermore, the YTHDF1 gene copy number was positively correlated with YTHDF1 mRNA expression in CRC. Knocking down the expression of YTHDF1 significantly inhibited the CRC cell's tumorigenicity *in vitro* and murine xenograft tumor growth *in vivo*. Furthermore, silencing of YTHDF1 inhibited the colonosphere formation ability *in vitro*. Mechanistically, we found that silencing YTHDF1 significantly inhibited Wnt/β-catenin pathway activity in CRC cells. Together, YTHDF1 is overexpressed in CRC and plays a vital oncogenic role in CRC, and this novel finding may provide a potential therapeutic target for CRC.

## Introduction

Colorectal cancer (CRC) is one of the most common malignant tumors in humans, with nearly 881,000 new deaths estimated to have occurred in 2018 worldwide, ranking second in cancer mortality (1). In China, the mortality and morbidity rates of colorectal cancer have demonstrated an increasing trend, ranking fifth among malignant tumors, with 376,000 new cases and 191,000 deaths each year (2). Although medical treatment is constantly improving, the 5-year relative survival rate for patients is only 64.9% (3). The main problem encountered during treatment is the resistance to therapy (4). Therefore, it is necessary to study the molecular biological mechanism of CRC.

Generally, cancer stem cells (CSCs) are thought to be closely related to the occurrence and development of tumors (5–7). The CSC hypothesis considers that many cancers are not mere monoclonal expansions of cells but might actually be akin to abnormal organs, sustained by a diseased CSC population (8). Additionally, recently, numerous studies have shown that CSCs play an important role in colorectal cancer (9–12). Therefore, to find and study the genes that regulate CRC cell stemness may be potential therapeutic approaches for CRC.

YTH N6-methyladenosine RNA binding protein 1 (YTHDF1) is a member of the YTH domain family, which includes YTHDF1, 2, and 3 and YTHDC1 and 2. In the cytosol, YTHDF1 functions as a “reader” of m6A-modified mRNAs and interacts with initiation factors to facilitate translation initiation (13). YTHDF2 regulates the degradation of m6A-modified mRNAs (14). YTHDF3 promotes protein synthesis in synergy with YTHDF1 and affects methylated mRNA decay mediated through YTHDF2 (15). However, the relationship between YTHDF1 and cancer is largely unknown. There are only a few reports on the relationship between YTHDF1 and tumors. Zhao et al. (16) reported the correlation between YTHDF1and hepatocellular carcinoma. Han et al. (17) reported that RNA m6A modification regulates anti-tumor immunity response via YTHDF1. And when we were doing this research, Nishizawa et al. (18) first reported that c-Myc promotes YTHDF1 expression, and YTHDF1 was associated with proliferation and chemosensitivity in CRC. But their research does not explain the mechanism of YTHDF1 in CRC. In our study, we found that DNA copy number amplification has a certain relationship with YTHDF1 overexpression. Mechanistically, we found that silencing YTHDF1 significantly inhibited the activity of Wnt/β-catenin pathway in CRC cells.

## Materials and Methods

### Cell Culture and Clinical Material

The human CRC cell lines SW620 and SW480 were cultured in L15 medium (KeyGEN BioTECH, Nanjing, China) containing 10% fetal bovine serum (FBS; Biological Industries, Israel), and HCT116 and HT-29 were cultured in McCoy's 5A medium (KeyGEN BioTECH, Nanjing, China) containing 10% FBS. The human normal colon mucosal epithelial cell line NCM460 was maintained in McCoy's 5A medium containing 10% FBS. The cells were grown in a 5% CO2 cell culture incubator at 37°C. Clinical material were obtained from patients treated at the Third Xiangya Hospital of Central South University (Changsha China) with informed consent and approval of the Medical Ethics Central South University.

### RNA Extraction and Real-Time PCR

For real-time PCR analyses, total RNA was extracted by Trizol (Invitrogen) from CRC cells and tissue. And cDNA was synthesized using ReverTra Ace qPCR RT Master Mix with gDNA remover (TOYOBO, Japan) following the manufacturer's recommendations. qPCR assays were performed using KOD SYBR® qPCR Mix (TOYOBO, Janpan) in the LightCycler® 480II System (Roche) following the manufacturer's instructions. Treated samples were normalized to controls with the ΔΔCt formula using GAPDH as an endogenous control. The primers used in this study can be found in Supplemental Table 2.

### Immunohistochemistry

The tissue sections from CRC patients were embedded by paraffin. Tissue sections were deparaffinized and rehydrated. Blocked the tissue sections' endogenous peroxidase activity with 0.3% hydrogen peroxide for 20 min. Then the tissue sections were blocked in 10% BSA for 10 min, and incubated with anti-human YTHDF1 antibodies (1:100, Proteintech) at 4°C for 12 h. After that tumor sections were incubated in biotinylated secondary antibodies for 20 min at room temperature. Thereafter, the tissue sections were reacted with streptavidin-peroxidase conjugate for 10 min. Then added 3,3'-diaminobenzidine as the chromogen substrate. Images were captured using an inverted microscope system (Olympus, IX73, Japan).

### Copy Number Variation (CNV) Detection Using the AccuCopy® Assay

DNA was isolated from CRC tissues and cells using a Qiagen kit according to the manufacturer's instructions (Qiagen). the AccuCopy assay, which was developed based on multiplex competitive amplification by Genesky Biotechnologies (Shanghai, China), was used to assess the copy number value of YTHDF1 in selected tissues and cells. The basic molecular principle of AccuCopy was well-described by Du et al. (19). The primers used in this assay are listed in Supplemental Table 3.

### Lentiviral Vector and Transfection

The lentiviral shRNA targeting human YTHDF1 and lentivirus carrying empty vector were purchased from Shanghai GenePharma Co., Ltd. To generate stable lentivirus-transduced lines, cells were infected with virus and polybrene following the manufacturer's recommendations, and stable cell lines were selected with 4 μg/ml of puromycin treatment after 72 h of transfection. The efficiency in different cells was determined by qRT-PCR and the GFP intensity. The shRNA sequences are listed in Supplemental Table 2.

### Cell Proliferation Assays

The Cell Counting Kit-8 (CCK8) (Dojindo, Japan) kit and Cell-Light™ EdU Apollo®567 *in vitro* Imaging Kit (Guangzhou RiboBio, China) were used to observe the proliferation rate of cancer cells. For CCK8 assay, about 2000 HT29 cells and about 8000 SW480 cells were seeded in 96-well plates (Corning, USA), respectively. CCK8 (10:100) was added into each well at the time point of 24, 48, 72, and 96 h after the cells were seeded. The absorbance values (A450) were detected using an EnVision microplate reader (PerkinElmer). For EdU assay, totally 4,000 cells were seeded in 96-well plate and treat following the manufacture's recommendations. The images were captured using an inverted microscope system (Olympus, IX73, Japan).

### Flow Cytometric Analysis

Cell cycle analysis was measured by flow cytometry. In total, about twenty thousand cells labeled with propidium iodide (PI; Sigma-Aldrich, USA) were analyzed by using FACSCalibur flow cytometer (BD Biosciences). The proportions of G0/G1, S and G2/M cells were calculated and compared by using ModFit LT 3.1 software.

For cell surface staining, 1 × 10^6^ HT29 colonosphere cells or parental HT29 cells labeled with CD44-APC (BD biosciences) were treated following the manufacturer's recommendation. The results were analyzed by using FACSCalibur flow cytometer (BD Biosciences).

### Cell Invasion Assays

Cell invasion assays were performed by using the Transwell chamber (24-well; 8.0-μm pore membranes) (Corning, USA) coated with Matrigel (Corning, USA) for SW480 and Corning® BioCoat™ Matrigel® Invasion Chamber (8.0 μm PET, 24-well) for HCT116. About 2 × 10^5^ cells were seeded in the upper chamber with serum-free medium+0.5% bovine serum albumin (BSA). The chemoattractant used in the lower chamber was medium+10% FBS. After incubation for 24~30 h and removal of the non-invading cells by PBS, the invading cells were fixed with formaldehyde and stained with 0.5% crystal violet for 30 min at room temperature. The images were captured using an inverted microscope system (Olympus, IX73, Japan).

### Anchorage-Independent Growth Assays

Anchorage-independent growth assays were performed in 6-well plates (Corning, USA). The bottom layer was covered with 1.5 ml of 1.2% agar in medium supplemented with 10% FBS and was allowed to solidify. The next day, the cells were seeded on top in 1 ml of 0.6% agar in medium containing 10% FBS. The number and size of clones were counted after 20 days. The images were captured using an inverted microscope system (Olympus, IX73, Japan).

### CSC Self-Renewal and Differentiation

For the sphere formation assay, about 1,000 HT29 cells were cultured in 6-well ultralow cluster plates (Corning, USA) in serum-free DMEM/F12 (1:1) medium (Gibco) supplemented with 20 ng/ml epidermal growth factor (EGF) (PeproTech) and 10 ng/ml fibroblast growth factor 2 (bFGF) (PeproTech), 1 × B27 supplement (Gibco), 2 μg/ml of 0.2% heparin (Solarbio) and 1% penicillin-streptomycin (P/S) for 8 days. For multilineage differentiation assay, the colonospheres of CSCs were plated on 24-well plastic plates (Corning, USA) in McCoy's 5A medium containing 8% fetal bovine serum (FBS) without growth factors for 48 h. The images were captured using an inverted microscope system (Olympus, IX73, Japan).

### Western Blotting

Cells and tissue were collected and lysed with RIPA lysis buffer supplemented with PMSF protease inhibitor (1:100) to harvest proteins. Then equal amounts of protein samples were resolved by 10% sodium dodecyl sulfate-polyacrylamide gel electrophoresis (SDS-PAGE), and then transferred to polyvinylidene fluoride membranes (PVDF).The PVDF membranes were washed with TBS and blocked with TBST containing 5% w/v skimmed milk. Thereafter, the PVDF membranes were incubated with primary antibody at 4°C overnight. The antibodies used for western blot are as follows: YTHDF1 (Protein Tech, 1:1000), FZD9 (ProteinTech, 1:1000 dilution), WNT6 (ProteinTech, 1:1000 dilution), non-phospho (active)-β-catenin (Cell Signaling Technology, #8814, 1:1000 dilution), GAPDH (ProteinTech, 1:2000). After washing with TBST, the PVDF membranes were incubated with horseradish peroxidase (HRP)-conjugated secondary antibody. Enhanced chemiluminescence system reagent (KeyGEN BioTECH, Nanjing, China) was used to visualize the bands.

### Immunofluorescence

Immunofluorescence (IF) was performed using the Immunol Fluorescence Staining Kit with kFluor555-Labeled Goat Anti-Rabbit IgG (KeyGen BioTECH) according to the manufacturer's instructions. Briefly, A total of 1.5 × 105 cells was plated into the 24-well plates that was covered by a glass coverslip. Cells were fixed in 4% paraformaldehyde for 30 min at room temperature and blocked in 5% BSA for 60 min at room temperature. Cells were labeled with anti-beta-catenin antibodies (1:100, Cell Signaling Technology #8480) at 4 °C overnight. The following day, cells were stained for 60 min with kFluor594-Labeled Goat Anti-Rabbit IgG (1:100;KeyGEN BioTECH, Nanjing, China) at room temperature. Nuclei were stained with DAPI (KeyGEN BioTECH) for 5 min at room temperature. The images were captured using a fluorescence microscope (Zeiss (ZEISS) LSM800 confocal microscope).

### Luciferase Reporter Assay

The TOPflash (β-catenin-binding TCF Reporter Plasmid) and FOPflash (mutated TCF binding site) plasmids were purchased from Shanghai Genechem Co., LTD. For the TOP/FOP luciferase assay, the cells were seeded in 24-well plates (Corning, USA) in triplicate. The indicated plasmids and pRL-TK (Promega) renilla plasmid were transfected into the cells by using the Lipofectamine 2000 (Invitrogen) according to the manufacturer's recommendations. Forty-eight hours after transfection, dual-luciferase reporter assays were performed according to the protocol using the Dual-Luciferase® Reporter Assay System (cat. E1910, Promega). The luciferase activity of reporter plasmids was normalized to the Renilla luciferase activity.

### RIP Assay

RNA-binding protein immunoprecipitation (RIP) was performed using the EZ-Magna RIP Kit (Merck, KGaA, Darmstadt, Germany, Catalog No. 17-701) according to the manufacturer's instructions. Briefly, about 2 × 10^7^ HT29 cells were washed with ice-cold PBS and resuspended with RIP lysis buffer containing protease inhibitor mixture and RNase inhibitor. Then, Magnetic Beads Protein A/G was incubated with 5 μg IgG (negative control) (Merck KGaA) or YTHDF1 (Proteintech) antibody for 30 min at room temperature. Then, the complexes were added cell lysis buffer and immunoprecipitation buffer with EDTA and RNase inhibitor. Thereafter, the complexes were incubated in rotator overnight at 4°C. The next day, the complex was washed with washing buffer containing with proteinase K and 10%SDS and then heated at 55°C for 30 min. Finally, RNA was extracted and purified for RT-qPCR analysis. RIP assays were performed in biological triplicates and were detected by RT-qPCR (the primers are described in Supplemental Table 2).

### Tumor Xenografts

All animal experimental procedures used in this study were approved by the Animal Ethics Committee of Central South University. Female BALB/c nude mice (4–5 weeks, 18–20 g) purchased from the Department of Laboratory Animals of Central South University were maintained under specific pathogen-free conditions. Respectively, 1 × 10^5^ parental HT29-shNC cells, HT29-shYTHDF1 cells, HT29-shNC colonospheres, and HT29-shYTHDF1 colonospheres were inoculated subcutaneously into the left inguinal folds of the nude mice. The minimum width (W) and maximum length (L) of the tumor were measured using a caliper every 4 days. After 6~7 weeks, all the mice were sacrificed, and all the organs were removed for examination. The tumors were stored in liquid nitrogen or embedded in paraffin for hematoxylin-eosin H&E staining.

### Statistical Analysis

Statistical computations were performed using GraphPad Prism 7. To compare the differences between two groups, Student's *t*-test was performed. The difference between the growth rates was determined by ANOVA with repeated measures analysis of variances. The correlation between the YTHDF1 gene copy number and mRNA expression was analyzed using Spearman's rank correlation. Box-whisker plots depict the mean, 1st and 3rd quartiles and min/max, and scatter plots depict the mean with or without SD. Statistical significance was analyzed by two-tailed unpaired Student's *t*-test, and *p* < 0.05 was considered statistically significant.

## Result

### YTHDF1 Is Overexpressed in CRC

**Figure 1:**
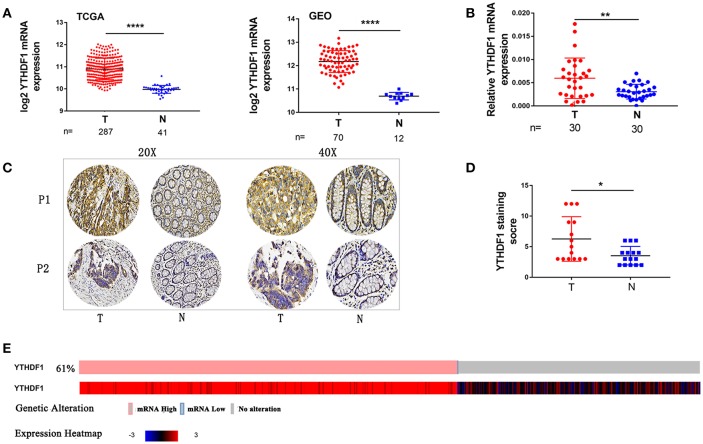
YTHDF1 Is Overexpressed In CRC. **(A)** YTHDF1 mRNA levels in the TCGA (left) and GEO (right) databases. **(B)** YTHDF1 mRNA expression in CRC tissues (T) and adjacent normal tissue (N). **(C)** Representative IHC images of YTHDF1 protein expression in CRC tissues (T) and adjacent normal tissue (N) of two patients (P1–P2). **(D)** Quantification of the IHC score of YTHDF1 in CRC tissue (T) and adjacent normal tissue (N). **(E)** 61%(321/524) CRC patients/samples with the increase in YTHDF1 expression in TCGA. (^*^p < 0.05; ^**^p < 0.01; ^***^p < 0.001; ^****^p < 0.0001).

To identify genes that are associated with CRC, we analyzed the expression of YTHDF1 in CRC on online databases The cancer genome atlas (TCGA) and Gene Expression Omnibus (GEO) profiling showed that YTHDF1 is significant upregulated in tumors compared with that in adjacent normal tissues in CRC (Figure 1A). Then, we validated that YTHDF1 is overexpressed at the mRNA level in 30 primary CRC tissue compared with adjacent normal tissue (Figure 1B and Figure S1) To further validate the expression level of YTHDF1, we detected the expression of YTHDF1 protein in 15 colon cancers and adjacent tissues by immunohistochemistry (IHC). The results showed that YTHDF1 protein expression level is higher in CRC tissue than adjacent tissue (Figures 1C,D). Additionally, we used the cBio Cancer Genomics Portal (20) to analyze the expression of YTHDF1 in CRC patients/samples, the result found that overexpression of YTHDF1 occurred in 61%(321/524)(Figure 1E). Furthermore, statistical analysis of the 30 specimens revealed that YTHDF1 expression was associated with tumor depth (*p* = 0.014) and tumor size (*p* = 0.028) (Supplemental Table 1).

### DNA Copy Number Amplification Contributes to YTHDF1 Overexpression in CRC

**Figure 2:**
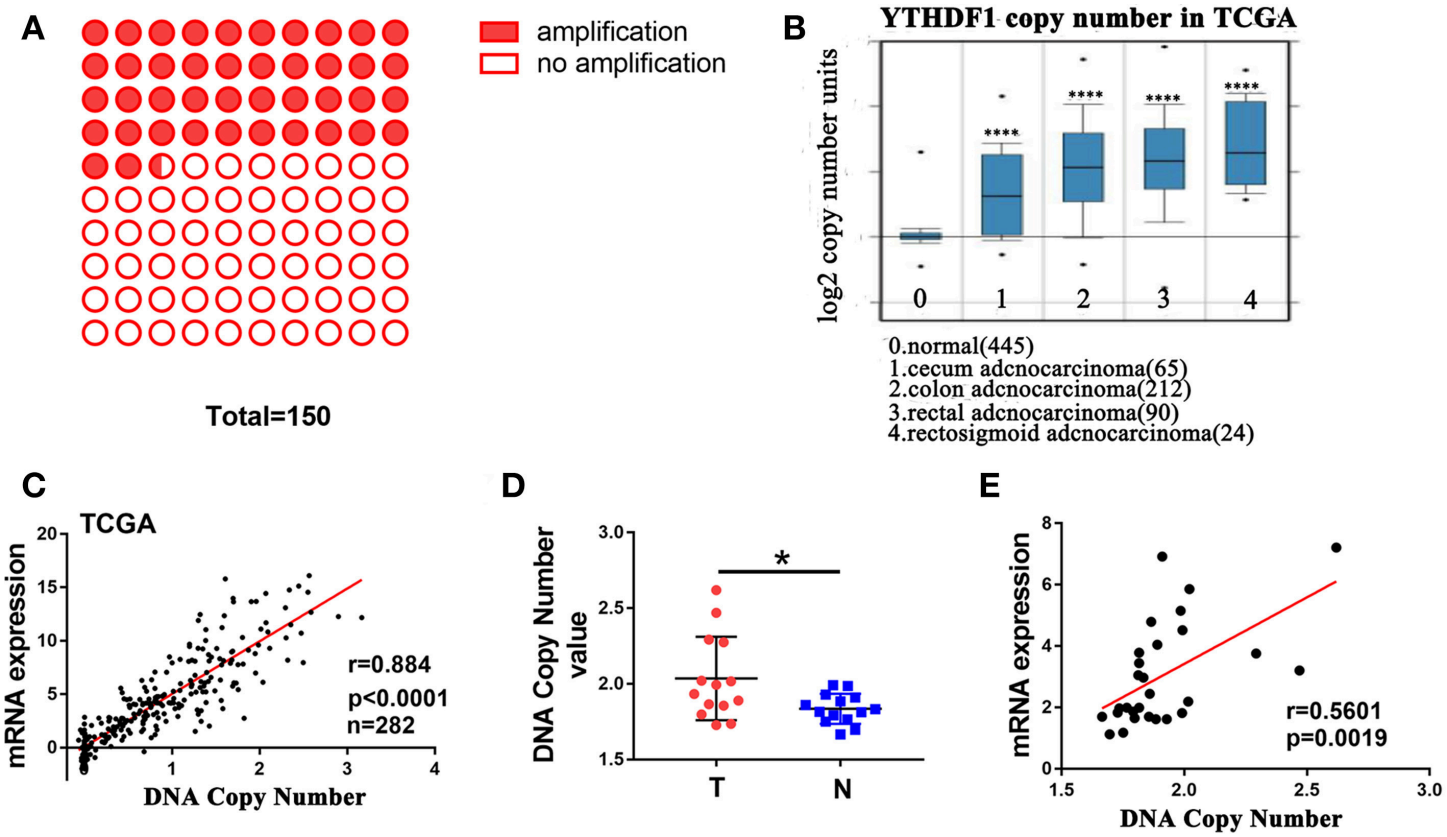
DNA copy number amplification contributes to YTHDF1 overexpression in CRC. **(A)** 42% (64/150) CRC patients with increased YTHDF1 expression had an amplification of the YTHDF1 copy number. **(B)** YTHDF1 copy numbers in TCGA CRC samples. **(C)** Correlation between the DNA copy number and mRNA expression of YTHDF1 in TCGA. **(D)** the comparison of the YTHDF1 DNA copy number between CRC tissues (T) and adjacent normal tissues (N) in 14 patients. **(E)** the correlation between the DNA copy number and mRNA expression of YTHDF1 in 14 pairs of CRC tissue. (^*^p < 0.05; ^****^ *p* < 0.0001).

A recent study reported that genes with CNAs are potential biomarkers and/or therapeutic targets for CRC (21). Additionally, The Catalog Of Somatic Mutations In Cancer (COSMIC) (22) showed that among 150 CRC patients with the increase in YTHDF1 expression, 42% (64/150) had an amplification of the YTHDF1 copy number (Figure 2A). These findings prompted us to speculate that CNAs may be related to the upregulation of YTHDF1. After analyzing the data from the ONCOMINE (23), we found that YTHDF1 copy number in CRC tumor were significant higher than that in normal tissue (blood, colon and rectum) (Figure 2B). Furthermore, YTHDF1 mRNA expression was significantly correlated with the copy number in TCGA-CRC samples (Figure 2C). To further validate the correlation between the mRNA expression and copy number, we used the AccuCopy copy number assay to examine 14 pairs of primary CRC tissue compared with adjacent normal tissue. We found that the YTHDF1 copy number in tumor tissue were higher than that in adjacent normal tissue (Figure 2D). Additionally, YTHDF1 mRNA expression was correlated with the copy number (Figure 2E). Therefore, our results suggest that copy number gain is one of a major mechanism that contributes to the overexpression of YTHDF1 in CRC.

### YTHDF1 Promotes CRC Cell Tumorigenicity *in vitro*

**Figure 3:**
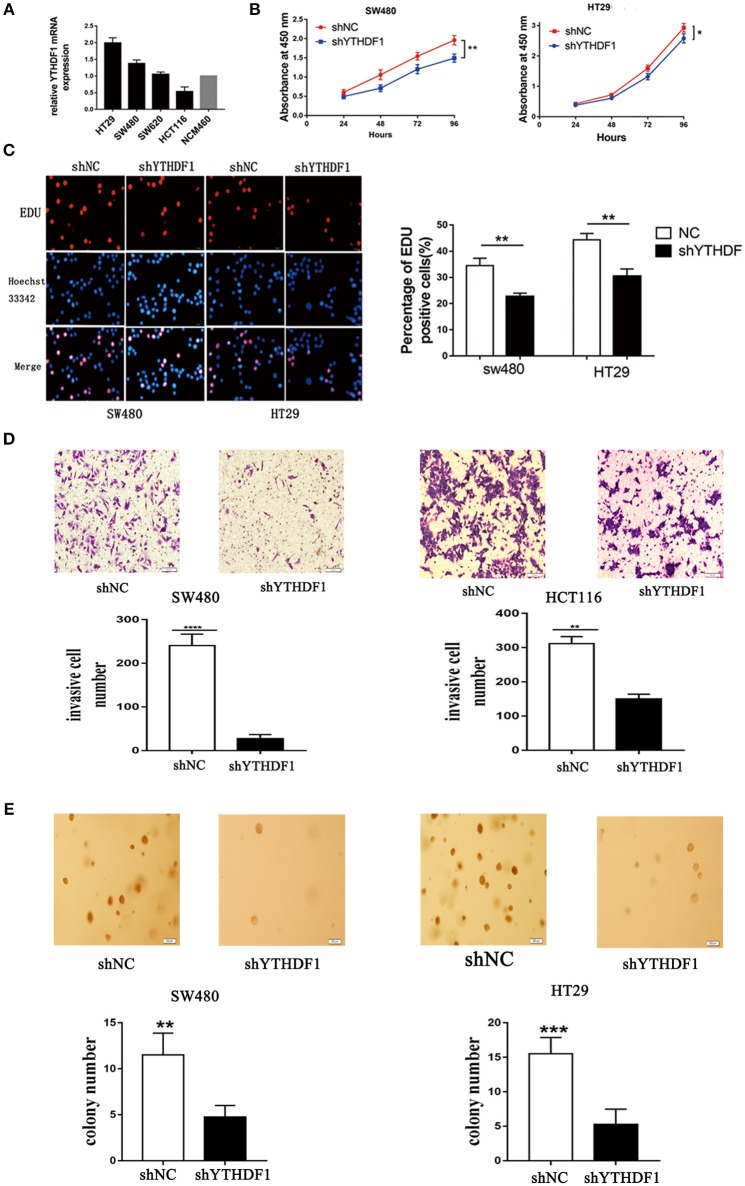
YTHDF1 Regulates CRC Cell Tumorigenicity *in vitro*. **(A)** mRNA expression of YTHDF1 in four CRC cell lines (HT29, SW480, SW620, HCT116), and normal human colon epithelial cell line (NCM460). **(B)** Knockdown of YTHDF1 significantly reduced cell proliferation by CCK8 assay in SW480 and HT29. **(C)** Representative images of Edu assay (scale bar, 20 um;) (left), and the ratio of EdU positive cells to total Hoechst33342 positive cells (right). **(D)** Representative images of the Matrigel invasion assay implying that knockdown of YTHDF1 reduce SW480 and HCT116 cell invasion (scale bar, 100 μm). **(E)** Representative image of the anchorage-independent growth assays implaying that knockdown of YTHDF1 reduced anchorage-independent growth of SW480 and HT29 cell (scale bar, 200 μm). (^*^p < 0.05; ^**^p < 0.01; ^***^p < 0.001; ^****^p < 0.0001).

We used reverse transcription-quantitative real-time PCR (RT-qPCR) to verify the expression of YTHDF1 in various CRC cell lines (HT29, HCT116, SW480, and SW620) (Figure 3A). To substantiate the function of YTHDF1 in CRC cells, we silenced the YTHDF1 expression in HT29 and SW480 cells with lentiviral vectors carrying YTHDF1-specific small hairpin RNAs (shYTHDF1). Control cells were transfected with lentiviral vectors carrying negative control shRNA (shNC). The transfection effect was observer by the green fluorescence protein expression, and the YTHDF1 silencing effect was confirmed byRT-qPCR and western blot (Figures S2A–C). First, we assessed the effect of YTHDF1 on cell proliferation using the Cell Counting Kit-8 (CCK-8) assay (Figure 3B) and Edu incorporation assay (Figure 3C). The results showed that YTHDF1 silencing reduced the cell proliferation activity. Additionally, we assessed the effect of YTHDF1 on invasive activity using the Transwell chamber assay and discovered that YTHDF1 silencing reduced the SW480 and HCT116 cell invasive activity (Figure 3D). Finally, we assessed the oncogenic potential of YTHDF1 *in vitro* using the anchorage-independent growth assay, and the results showed that knockdown YTHDF1 reduced anchorage-independent growth in CRC cells (Figure 3E). Collectively, these results suggest that YTHDF1 plays a pivotal oncogenic role in CRC cells *in vitro*.

### YTHDF1 Promotes the Cell Cycle Progression of CRC Cell

**Figure 4:**
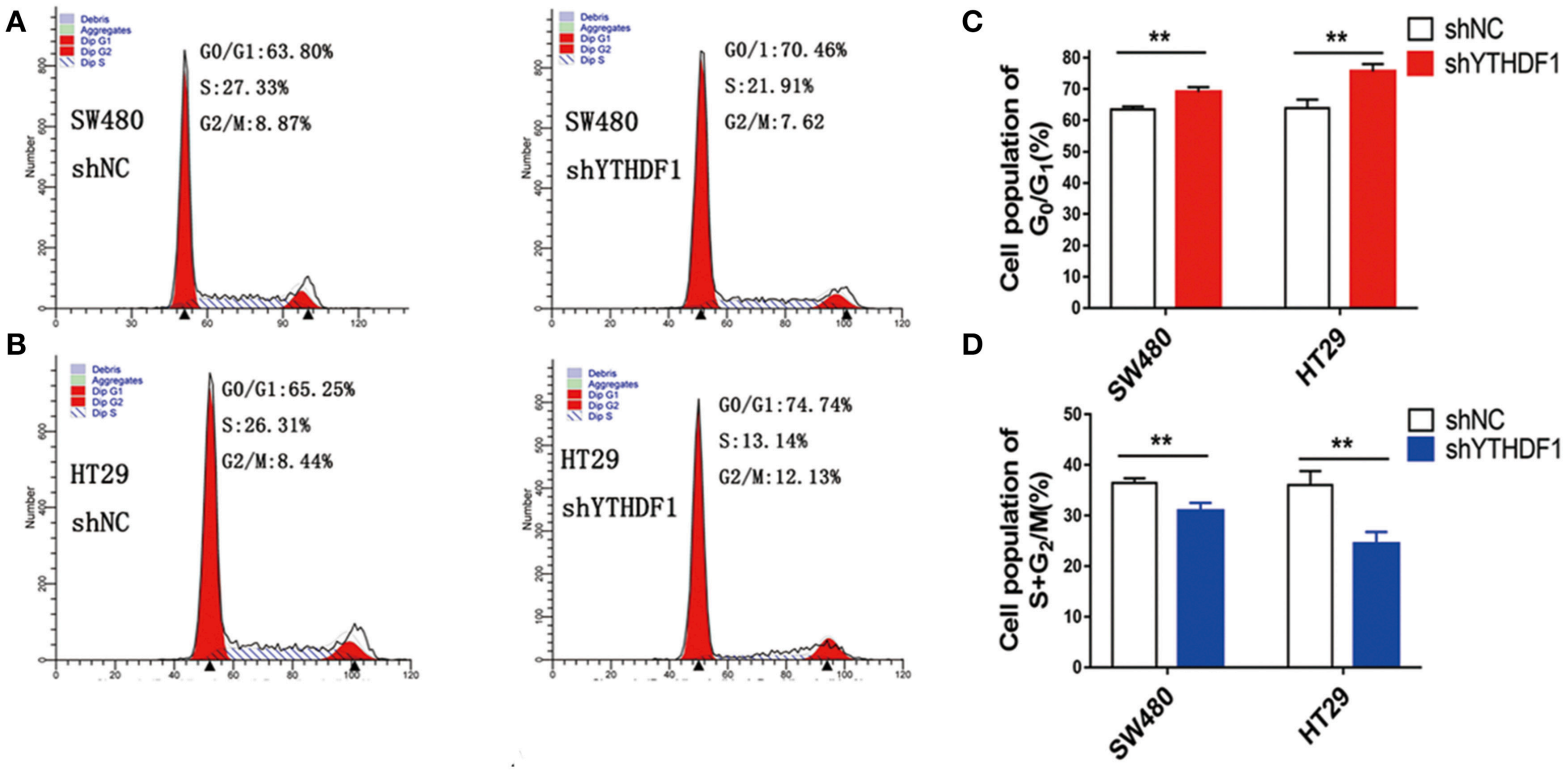
YTHDF1 promotes the cell cycle progression of CRC cell by flow cytometry analysis. Knockdown of YTHDF1 increased the G0/G1 phase fraction and decreased the S-phase fraction. **(A)** SW480 cells with shNC transfection (left panel), with shYTHDF1 transfection, **(B)** HT29 cells with shNC transfection (left panel), with shYTHDF1 transfection. **(C)** The statistics of SW480 cells with shNC or shYTHDF1 transfection. **(D)** The statistics of HT29 cells with shNC or shYTHDF1 transfection. (^**^ *p* < 0.01).

As YTHDF1 regulates CRC cell proliferation, we assessed whether the YTHDF1 impacts on the cell cycle progression. As expected, the results showed that silencing the YTHDF1 expression caused more cells to be arrested in the G1 phase and fewer cells to be arrested in the S phase (Figures 4A–D). These results suggested that YTHDF1 regulates the cell proliferation partly due to its impact on the cell cycle progression.

### YTHDF1 Is Overexpressed in Colonospheres and Regulates Stem Cell-Like Activity

**Figure 5:**
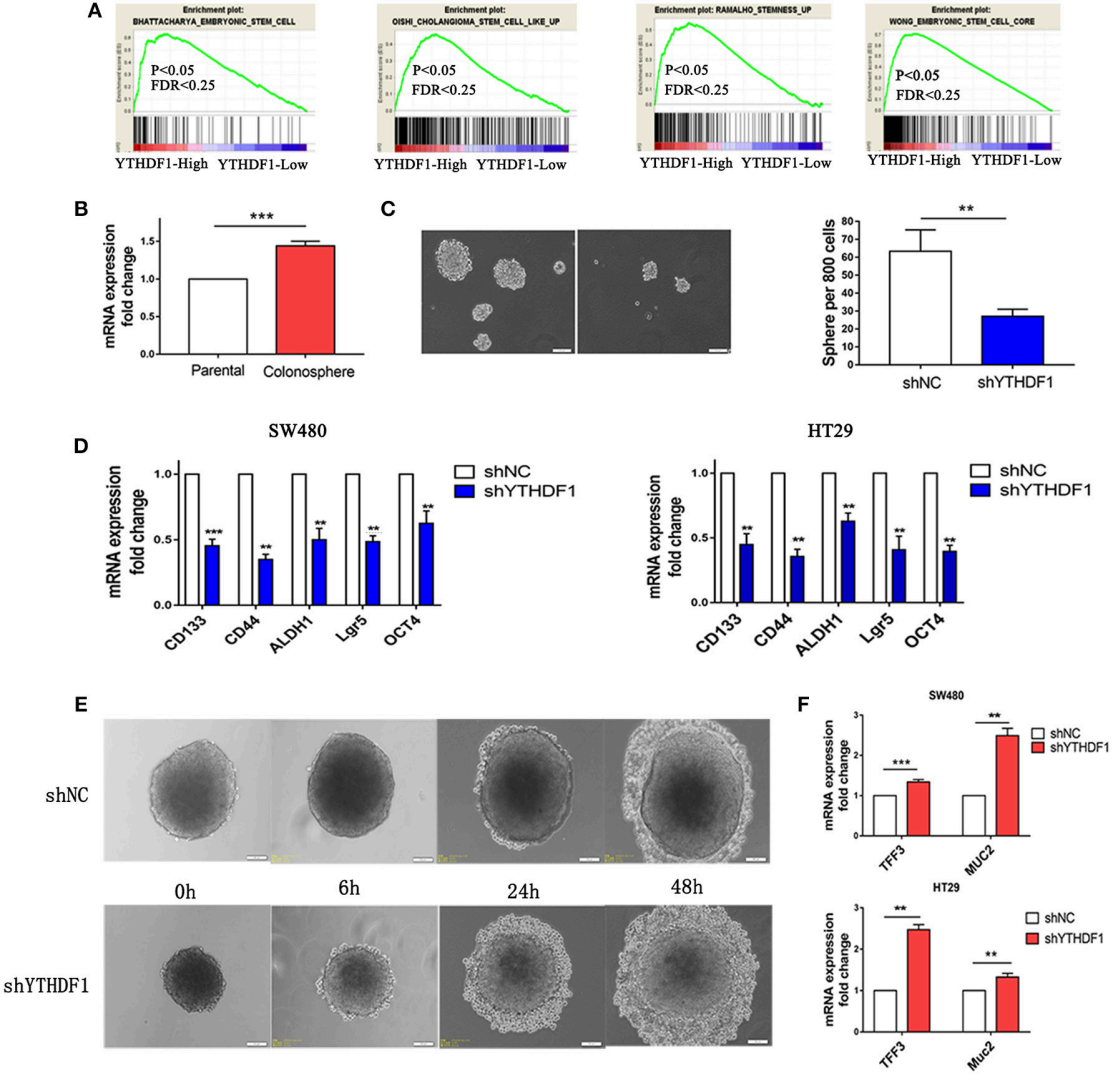
YTHDF1 Is Overexpressed in Colonospheres and Regulates Stem cell-like activity. **(A)** GSEA plot showing YTHDF1 expression level was positively correlated with activated stemness related gene signatures **(B)** Real-time PCR analysis the YTHDF1 expression between parental HT29 cell and HT29 colonospheres cells **(C)** Representative images and quantification of spheres formed by HT29 cells (scale bar, 100 μm;). **(D)** Real-time PCR analysis of stemness-related markers in CRC cells. **(E)** Representative images of HT29 colonospheres cultured under differentiation-inducing conditions. (scale bar, 100 μm;) **(F)** Real-time PCR analysis of enterocyte markers in CRC cells (^**^p < 0.01; ^***^p < 0.001).

When we study the GEO database, we found that YTHDF1 was overexpressed in the HT29 colonospheres as compared with parental HT29 cells (Figure S3A GSE14773). Then we performed a gene set enrichment analysis (GSEA) using mRNA expression data from the GEO database and discovered that YTHDF1 expression was positively associated with stemness signatures in the CRC patient expression profiles (GSE32323; Figure 5A). Colonospheres could expand from HT29 cells when cultured under bFGF (+) EGF (+) serum-free conditions (24–26). To further validate that HT29 colonospheres are stem-like spheroid cells, we tested the expression level of CD44 (Figure S3B), which is a putative marker of CRC cancer stem cells (27). Additionally, we used RT-qPCR to validate YTHDF1 expression in colonospheres compared with that in parental HT29 cells. The results are consistent with the data from the GEO Profiles that YTHDF1 is overexpressed in colonospheres (Figure 5B). This finding prompted us to speculate that YTHDF1 may exert its tumorigenic effects, in part, by promoting stem cell-like activity. Previous studies have demonstrated that malignant cells have more stemness features, mainly to enhance the self-renewal capacity and inhibit differentiation (28–30). First, we assessed the impact of YTHDF1 on the self-renewal capacity by the sphere-forming assay. We found that YTHDF1 silencing significantly reduced the number of colonospheres (Figure 5C). Next, we validated whether YTHDF1 silencing could modulate CRC cancer stem cell markers, including CD44, CD133, OCT4, ALDH1, and Lgr5 (27). The results showed that YTHDF1 silencing significantly downregulated the expression of CRC cancer stem cell markers (Figure 5D). When colonospheres were cultured under differentiation-inducing conditions, colonospheres with silenced YTHDF1 expression showed proliferation of more epithelioid morphological cells than the control (Figure 5E). Additionally, we used RT-qPCR to verify the expression of YTHDF1 on enterocyte markers, including TFF3 and Muc2 (31). The results showed that YTHDF1 silencing upregulated the enterocyte markers (Figure 5F). Overall, these results suggest that YTHDF1 plays a pivotal role in stem cell-like activity.

### YTHDF1 Promotes Tumorigenicity of CRC Cells *in vivo*

**Figure 6:**
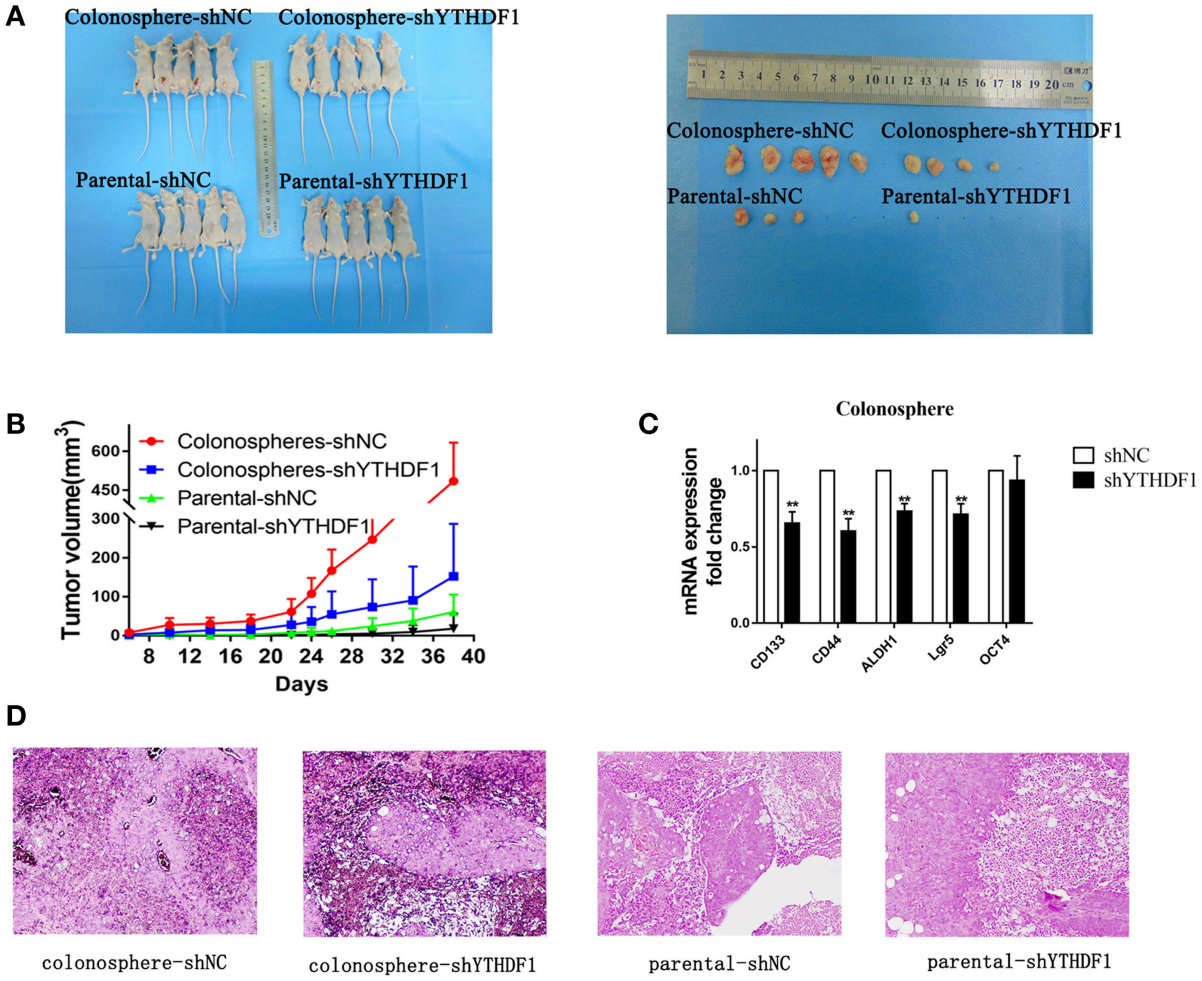
YTHDF1 Promotes the Tumorigenicity of CRC cells *in vivo*. **(A)** Imaging of mice (left) and xenograft tumors (right) showed that YTHDF1 silencing led to smaller tumors and colonospheres formed larger tumors. **(B)** Tumor formation growth curves. **(C)** Real-time PCR analysis of stemness-related markers in colonosphere xenograft tumors with shNC or shYTHDF1 transfection. **(D)** Representative images of H&E staining of mouse xenograft tumors (scale bar, 100 μm;) (^**^p < 0.01).

To further demonstrate the effect YTHDF1 on tumorigenicity, we subcutaneously injected 1 × 10^5^ parental HT29-shNC cells, HT29-shYTHDF1 cells, and HT29-shNC colonospheres, and HT29-shYTHDF1 colonospheres into the left inguinal folds of nude mice. The results showed that YTHDF1 silencing formed smaller tumors (Figures 6A,B).Then we validated the expression of CD133, CD44, ALDH1, OCT4, and Lgr5 in xenograft tumors by RT-qPCR, the results showed that YTHDF1 silencing downregulated the expression of CRC cancer stem cell markers in xenograft tumors (Figure 6C). Additionally, we used hematoxylin-eosin staining to stain the tissue removed from the nude mice (Figure 6D).

### Silencing YTHDF1 Inhibits the Wnt/β-Catenin Pathway in CRC Cells

**Figure 7:**
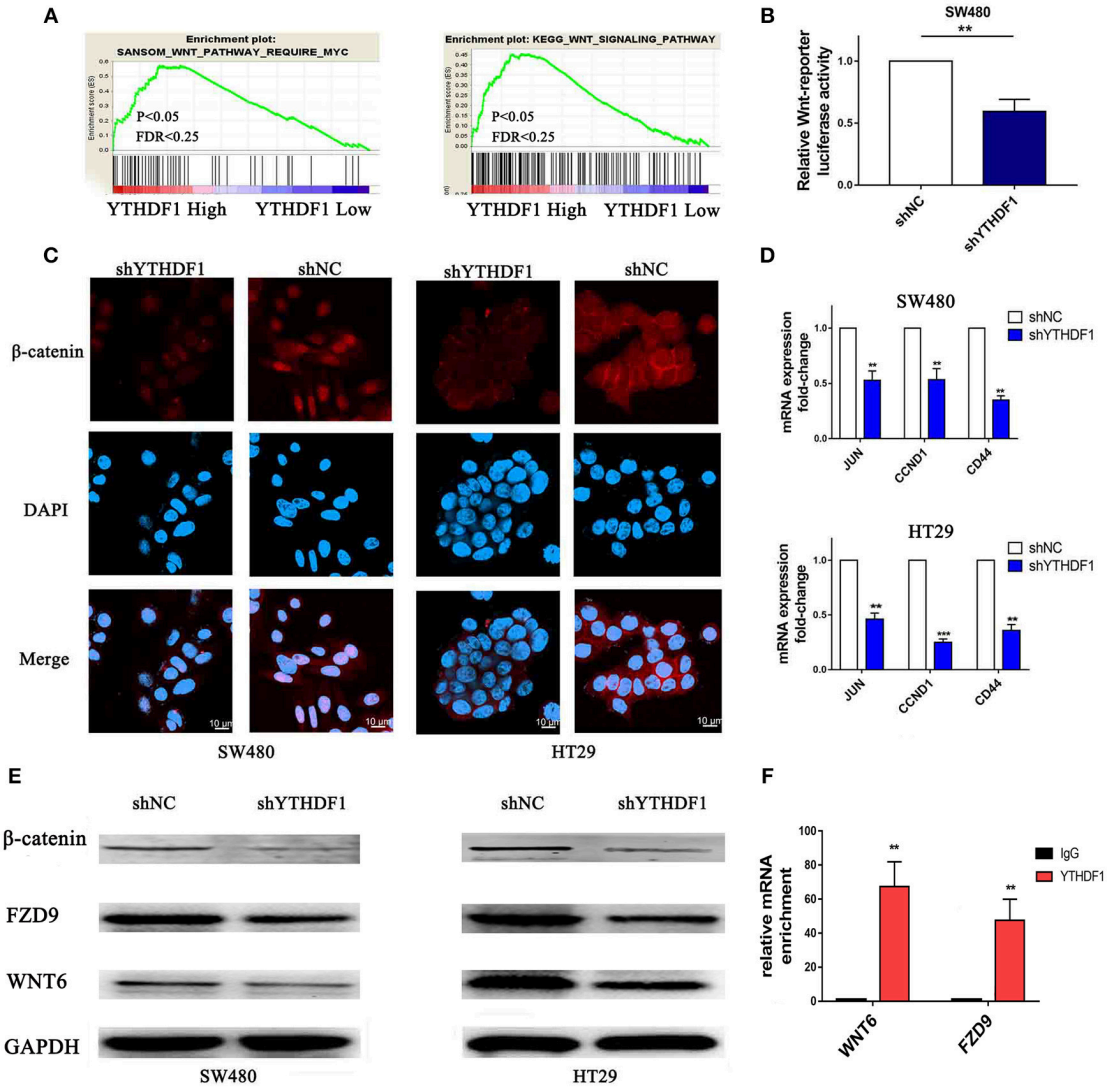
Silencing of YTHDF1 Inhibits the Wnt/β-Catenin Pathway in CRC Cells. **(A)** GSEA showing the positive correlations between YTHDF1 mRNA levels and Wnt signaling based on publicly available CRC profiles. **(B)** Luciferase-reporter assays of TOP/FOP transcriptional activity in the CRC cells **(C)** Immunofluorescence staining detected the protein expression and localization of β-catenin (scale bar, 10 μm;). **(D)** Real-time PCR of the mRNA expression of Wnt/β-catenin downstream genes (JUN, CCND1, CD44); **(E)** Western blotting analysis WNT6, FZD9 and non-phospho (active)-β-catenin protein expression in CRC cells. **(F)**. Real-time PCR of WNT6 and FZD9 enrichment of YTHDF1 compared with IgG. (^**^P < 0.01; ^***^P < 0.001).

To study the mechanism related to the pro-tumorigenic action of YTHDF1, we analyzed the expression of YTHDF1 and the genes regulated by various signaling signatures using GSEA of GEO and TCGA CRC datasets (GSE32323). We found that YTHDF1 mRNA expression correlated positively with Wnt-activated gene signatures (Figure 7A). Additionally, recent studies verified that the Wnt/β-catenin pathway plays an important role in stem cells and cancer stem cells (32–35). The above results indicated that the function of YTHDF1 may be related to the Wnt/β-catenin pathway. Furthermore, TOP/FOP flash assays validated that YTHDF1 silencing attenuated the reporter activity (Figure 7B). Meanwhile, we used immunofluorescence staining (Figure 7C) and Western blotting (Figure 7E) assay to assess β-catenin protein expression and localization. The results showed that YTHDF1 silencing reduced the nonphospho (active)-β-catenin expression and β-catenin nuclear signals activity. On the other hand, we detected the mRNA expression levels of Wnt/β-catenin downstream targets, including c-JUN, CCND1, and CD44. The results showed that YTHDF1 silencing downregulated the expression of these genes (Figure 7D). To further explore the direct interaction between the YTHDF1 and Wnt signal pathway, we analysis the Gene Ontology analysis of target genes identified by PAR-CLIP and RIP-seq for YTHDF1 that reported by Wang et al (13). We found that Wnt signal components FZD9 and WNT6 were located therein. Then we used western blot to assess the FZD9 and WNT6 protein expression in CRC cells after silencing YTHDF1 (Figure 7E). The results showed that YTHDF1 silencing downregulates the expression of these genes. Then we used RIP assay to assess the directly interact between the WNT6 and FZD9 with YTHDF1 in HT29 CRC cells, the results showed that WNT6 and FZD9 mRNA was linked by YTHDF1 protein (Figure 7F). Overall, these data suggest that the YTHDF1 regulated the Wnt/β-Catenin pathway activity in CRC Cells.

## Discussion

YTHDF1 gene is localized on chromosome 20q11. Previous studies have reported that gene overexpression is commonly found in CRC (36), and DNA copy number gain is an important cause that drives aberrant overexpression of oncogenes in cancer (37–39). In this study, we analyzed the TCGA and GEO online databases. The results showed that YTHDF1 mRNA is upregulated in CRC, and this high expression is associated with the DNA copy number. Our results with the clinical CRC specimens were similar to the online data, in which YTHDF1 was overexpressed in CRC at both the mRNA and protein levels, and the gain of copy number may be a major mechanism that contributes to the overexpression of YTHDF1 in CRC. Furthermore, we found the YTHDF1 expression level was correlated with clinical statistical, such as tumor depth and tumor size. Due to our biological tissues were only 30 pairs, our clinical analysis date have certain limitations, thus further clinical trials in a multicenter are needed.

YTHDF1 serves as a “reader” of m6A-modified mRNAs (13). Recent studies have shown that m6A is closely related to multiple tumors. m6A promotes the proliferation and tumorigenicity of endometrial cancer(40). The m6A methyltransferase METTL3 is upregulated in lung cancer and is required for the growth, survival, and invasion of cancer cells (41). As a m^6^A RNA demethylase, FTO is highly expressed in acute myeloid leukemia (42). Meanwhile, m6A plays an important role in regulating the characteristics of stem cells and cancer stem cells (43–45). Additionally, the online database showed that the YTHDF1 gene has a cancer-promoting potential score of B, while its knockdown markedly suppressed xenograft tumor growth in a mouse model. When we knocked down YTHDF1, it significantly inhibited CRC cell tumorigenicity *in vitro* and *in vivo*. Furthermore, knockdown of YTHDF1 inhibited colonosphere self-renewal but enhanced differentiation. These results suggest that YTHDF1 plays an important role in tumorigenicity and stem cell-like activity in CRC cells, which may be expected for the development of novel therapeutic treatments.

Wnt signaling plays an important role in the normal embryonal development of different tissues and regulates growth apoptosis and differentiation (46, 47). Wnt pathway component alterations can lead to CRC (46). Additionally, the Wnt/β-catenin pathway is related to cancer stem cells (34, 47). Mei et al. demonstrated that replication timing regulatory factor-1 through the Wnt/β-catenin pathway promotes stem cell-like traits in non-small cell lung cancer (48). Song et al. reported that transcription factor AP-4 regulates the tumor-initiating cell-like phenotype by activating the Wnt/β-catenin pathway in hepatocellular carcinoma cells (49). Furthermore, Giacomo Lettini et al. and Paloma Ordonez-Moran et al. demonstrated that the Wnt/β-catenin pathway participates in regulating the stem cell-like activity in CRC cells (11, 12). Our results showed that knock down of YTHDF1 inhibited the activity of the Wnt/β-catenin pathway, and we confirmed that YTHDF1 protein interacts with WNT6 and FZD9 mRNA. These results suggest that YTHDF1 regulates tumorigenicity and stem cell-like activity in CRC cells via the Wnt/β-catenin pathway. YTHDF1 could control mRNA translation via selectively recognizing and promoting ribosome loading of m6A-modified mRNAs (13). We propose that YTHDF1 recognizes and promotes the translation of m6A-modified FZD9 and Wnt6 mRNA, leading to aberrant activation of Wnt/β-catenin signaling and ultimately affecting the tumorigenicity and stem cell-like activity in CRC. However, in this study, we did not verify whether there are abnormal changes in the m6A-modified mRNA level in CRC and whether these genes mRNA are modified by m6A in CRC. These issues require further investigation in future research.

We demonstrated, for the first time, that copy number gain is a major mechanism that contributes to the overexpression of YTHDF1 in CRC. Additionally, we identified the oncogenic role of YTHDF1 in CRC. Knockdown of YTHDF1 downregulates FZD9 and WNT6 expression and inhibits the activity of the Wnt/β-catenin pathway. Although the underlying mechanism of the specific interaction between YTHDF1 and FZD9 and WNT6 needs to be clarified in future research, this discovery indicates that YTHDF1 regulates tumorigenicity and stem cell-like activity in CRC and may provide a potential therapeutic target for CRC.

## Ethics Statement

This study was carried out in accordance with the recommendations of Medical Ethics Central South University' with written informed consent from all subjects. All subjects gave written informed consent in accordance with the Declaration of Helsinki. The protocol was approved by the Medical Ethics Central South University.

## Author Contributions

YB, DL, and YZ conceived and designed the study. YB analyzed the data and prepared the manuscript. YB and CY performed *in vitro* and *in vivo* experiments. RW and CL participated in study design. SS, PY, and WL collected clinical specimens and information. LH designed the primer for qRT-PCR. All authors read and approved the final manuscript.

### Conflict of Interest Statement

The authors declare that the research was conducted in the absence of any commercial or financial relationships that could be construed as a potential conflict of interest.
