# Supplementary material for: YTHDF1 Regulates Tumorigenicity and Cancer Stem Cell-Like Activity in Human Colorectal Carcinoma
Source: Front Oncol. 2019 May 3;9:332. doi: 10.3389/fonc.2019.00332 (PMC6509179; doi:10.3389/fonc.2019.00332)
Supplement: Supplementary file 1 [file Data_Sheet_1.docx]

**Supplement Table1. Clinicopathologic characteristics and YTHDF1 expression of CRC patients**

| Characteristic | | YTHDF1 expression level chi-square test | | |
| --- | --- | --- | --- | --- |
|  |  | High(15) Low(15) P value | | |
| Age (years) | ≤60 | 9 | 8 | 0.7125 |
|  | >60 | 6 | 7 |  |
| Gender | Male | 11 | 9 | 0.4386 |
|  | Female | 4 | 6 |  |
| Depth | Tis/T1/T2 | 0 | 6 | 0.0143 |
|  | T3/T4 | 15 | 9 |  |
| Lymph node metasis | N0 | 6 | 10 | 0.2410 |
|  | N1/N2.N3 | 9 | 5 |  |
| Distant metastasis | M0 | 12 | 11 | 0.7505 |
|  | M1 | 3 | 2 |  |
| Stage | Ⅰ-Ⅱ | 4 | 8 | 0.1360 |
|  | Ⅲ-Ⅳ | 11 | 7 |  |
| Tumor size(cm) | <5 | 4 | 10 | 0.0281 |
|  | >5 | 11 | 5 |  |
| CEA | -(<10ng/ml) | 9 | 11 | 0.4386 |
|  | +(>10ng/ml) | 6 | 4 |  |

**Supplemental Table S2 primers and shRNA seqence**

| **Sequence** | **Target** | **Sense/Forward** | **Antisense/Reverse** |
| --- | --- | --- | --- |
| shRNA | human YTHDF1 | CCGCGTCTAGTTGTTCATGAA | N/A |
| shRNA | human LV3-NC | TTCTCCGAACGTGTCACGT | N/A |
| qPCR primers | human YTHDF1 | ATACCTCACCACCTACGGACA | GTGCTGATAGATGTTGTTCCCC |
| qPCR primers | human CD133 | GGCCCAGTACAACACTACCAA | ATTCCGCCTCCTAGCACTGAA |
| qPCR primers | human CD44 | CTGCCGCTTTGCAGGTGTA | CATTGTGGGCAAGGTGCTATT |
| qPCR primers | human Lgr5 | CTTCCAACCTCAGCGTCTTC | TTTCCCGCAAGACGTAACTC |
| qPCR primers | human OCT4 | CAAAGCAGAAACCCTCGTGC | TCTCACTCGGTTCTCGATACTG |
| qPCR primers | human ALDH1 | CCGTGGCGTACTATGGATGC | GCAGCAGACGATCTCTTTCGAT |
| qPCR primers | human c-JUN | CCAACTCATGCTAACGCAGC | TCTCTCCGTCGCAACTTGTC |
| qPCR primers | human CCND1 | GCTGCGAAGTGGAAACCATC | CCTCCTTCTGCACACATTTGAA |
| qPCR primers | human WNT6 | GGTGCGAGAGTGCCAGTTC | CGTCTCCCGAATGTCCTGTT |
| qPCR primers | human GAPDH | CTGACTTCAACAGCGACACC | TTACTCCTTGGAGGCCATGT |
| qPCR primers | human FZD9 | CATGTGCTACAACGTCTACTCG | GTGGCCCCATTTCTTCCCG |

**Supplemental Table S3 Copy number experiment information**

| **NO** | **Gene fragment** | **chromosome** | **Amplification length (+28)** | **Remarks** | **position of the product（hg19）** | **F primer 5-3** | **R primer 5-3** |
| --- | --- | --- | --- | --- | --- | --- | --- |
| **1** | 2p | Chr2 | 75+28 | Reference gene | 84500611-84500685 | TGAGCCAAAAATTCAGAATACAAGGA | TTGCTTGGAAGGCAGGCAAAC |
| **2** | YTHDF1_2 | Chr20 | 85+28 |  | 61826960-61827044 | AAAATGGTGCCCATGCCTGAC | GCGTCTGCTCTCTAGGACGGTAA |
| **3** | 16p | Chr16 | 125+28 | Reference gene | 25258413-25258537 | GGGACAGGCCTGAAGTGTTTC | AGCAGCAGCAGTGGGGTTTAG |
| **4** | 10pL | Chr10 | 145+28 | Reference gene | 31120531-31120675 | CACTGAGCCCCAGAGACCTGAC | AATGCACACCTCCAGGGAAAAC |
| **5** | 6q | Chr6 | 166+28 | Reference gene | 70860452-70860617 | TTGAGCAACAGTGAAACCCCTTTA | GCCCAAACACTGTGGGAGGTAG |
| **6** | YTHDF1_1 | Chr20 | 180+28 |  | 61846783-61846962 | GCGGTGCAGAGAACAAAAGGAC | CAGAATCGTCGCACAACCTCAA |
| **7** | 20q | Chr20 | 222+28 | Reference gene | 35865921-35866142 | AGGGTGCTGGGATCAGAGAGAG | GCTACTGGAGGGTGGCAAAATG |

**Figure S1**

**
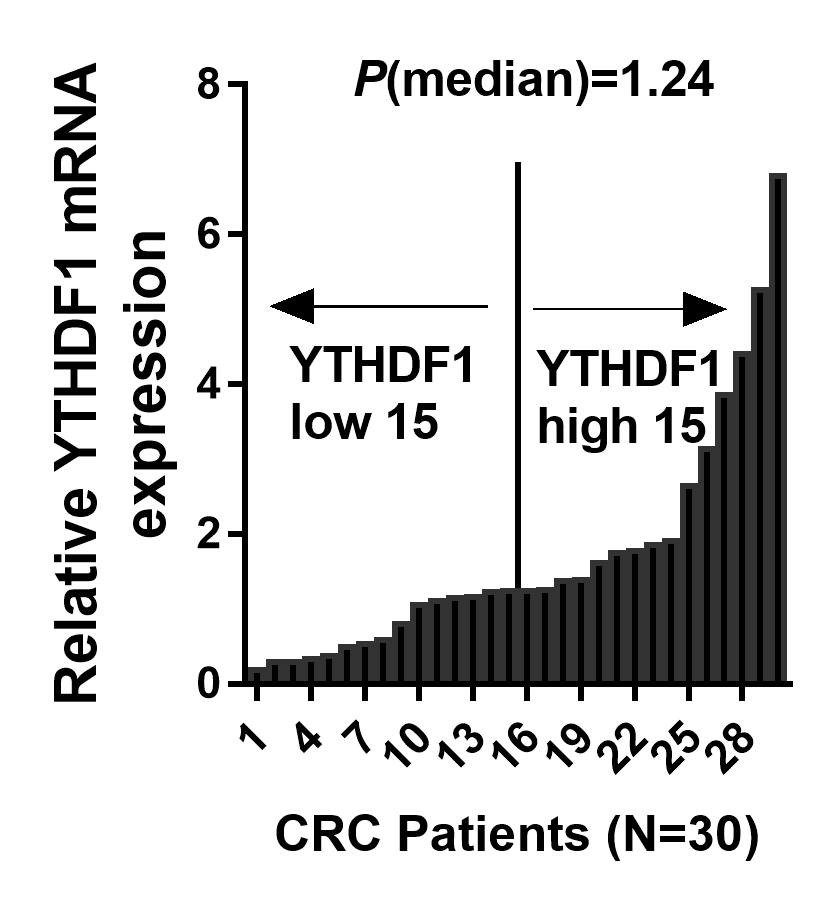
**

**Figure S2**

**

**

**Figure S3**

**
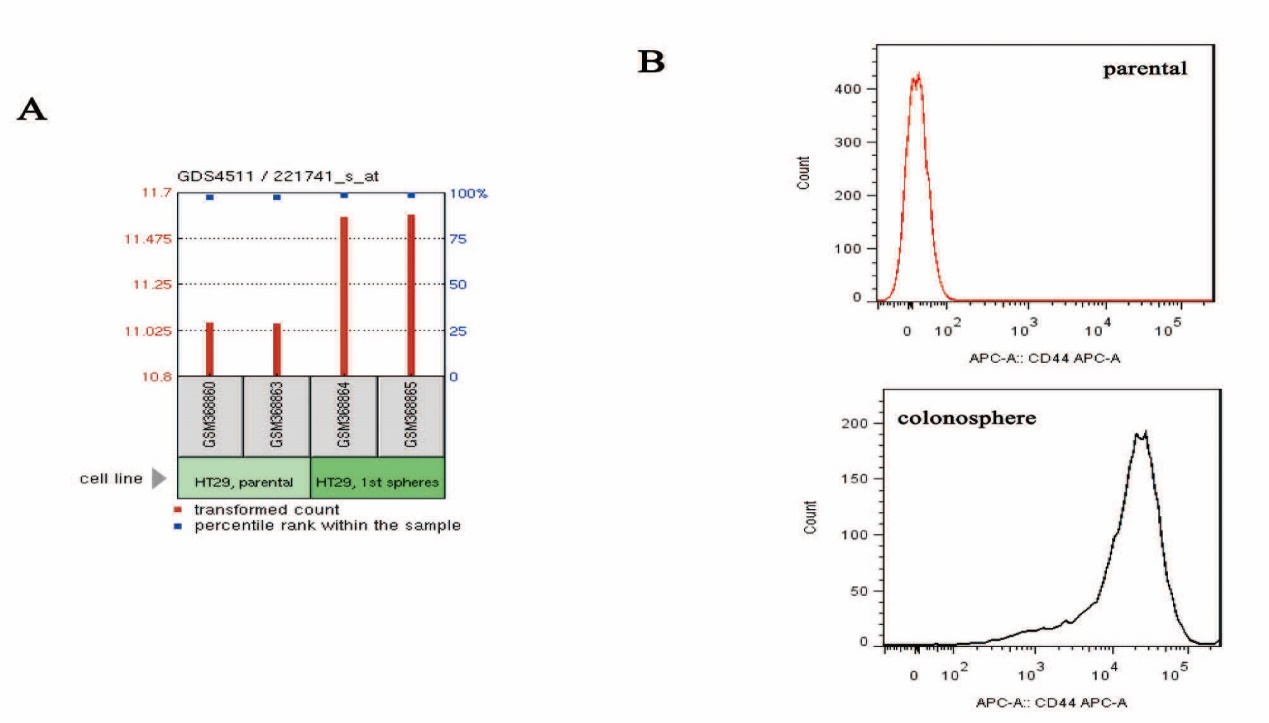
**

**Legends for Supplemental Figures**

**F**igure S1 Based on the median YTHDF1 mRNA expression level in the 30 pairs of tissues, we divided the patients into two subgroups with high or low tumor YTHDF1 expression levels

Figure S2 A. GFP in lentivirus transfected SW480 cell line and relative mRNA levels of YTHDF1 were measured by real time q-PCR assays after transfection of negative control (NC) or shYTHDF1. B. GFP in lentivirus transfected HT29 cell line and relative mRNA levels of YTHDF1 were measured by real ti-me q-PCR assays after transfection of negative control (shNC) or shYTHDF1. C. relative protein levels of YTHDF1 were measured by western blot(*p< 0.05, ***p < 0.01).

Figure S3 A. YTHDF1 mRNA expression level in HT29 colonosphere and HT29 parental in GEO DataSets (GSE63591). B. The CD44 protein expression level in HT29 colonosphere and HT29 parental measured by flow cytometric
